# Survival benefit of radical prostatectomy in bone metastatic prostate cancer stratified by disease characteristics: A SEER-based retrospective analysis

**DOI:** 10.1371/journal.pone.0326429

**Published:** 2025-06-27

**Authors:** Xinxing Zhang, Yuxuan Wang

**Affiliations:** 1 Chengdu New Radiomedicine Technology Co. Ltd., Chengdu, Sichuan, China; 2 Department of Nephrology, Chengdu Second People’s Hospital, Chengdu, Sichuan, China; Memorial Sloan Kettering Cancer Center, UNITED STATES OF AMERICA

## Abstract

**Background:**

The role of radical prostatectomy (RP) in patients with newly diagnosed bone-metastatic prostate cancer (PCa) remains insufficiently explored.

**Patients and methods:**

Patients with newly diagnosed bone-metastatic PCa were retrospectively identified from the SEER-17 database and categorized into two groups based on local treatment: biopsy-only and RP. Notably, patients who had received radiotherapy were excluded due to the unavailability of radiotherapy target site details in the SEER database, which made it impossible to determine whether the radiotherapy was directed at metastatic lesions or the prostate. Kaplan-Meier methods were used to estimate cancer-specific survival (CSS) and overall survival (OS) between the two groups. Subgroup analyses stratified by T stage, N stage, PSA levels, and ISUP grade were conducted to assess the impact of disease characteristics on the efficacy of RP. A risk score incorporating these disease characteristics (T stage, N stage, PSA level, ISUP grade) was assigned to each patient, and risk-stratified subgroup analyses were performed to further evaluate the relationship between the efficacy of RP and overall disease characteristics.

**Results:**

A total of 9,243 patients were included in this study, of whom 8,949 (96.8%) underwent biopsy alone and 294 (3.2%) underwent RP. Patients who underwent RP had better CSS (adjusted HR = 0.32, 95% CI: 0.23–0.44, p < 0.001; 5-year CSS rate: 83.0% vs. 44.5%) and OS (adjusted HR = 0.34, 95% CI: 0.26–0.45, p < 0.001; 5-year OS rate: 79.2% vs. 36.9%) compared with patients who underwent biopsy alone. The survival benefit persisted across all subgroups but were attenuated in patients with more advanced stage (T3 and N1) and higher grades of disease (PSA > 72.5 ng/ml and ISUP grade IV-V). Risk score analysis revealed diminishing benefits with increasing scores. Significant survival benefits were observed for scores 0–3 (all adjusted HR < 1, p < 0.05), whereas no survival differences were detected at the highest risk score (CSS: adjusted HR = 1.74, 95% CI: 0.54–5.65, p = 0.356; OS: adjusted HR = 1.56, 95% CI: 0.48–5.04, p = 0.456).

**Conclusion:**

Survival benefits of RP in de novo bone metastatic prostate cancer are modulated by disease characteristics, with attenuated effects in advanced/high-grade disease. Risk-stratified patient selection is critical, and prospective studies are needed to validate optimal candidacy for RP.

## Introduction

Prostate cancer (PCa) ranks as one of the most prevalent malignancies worldwide, representing the fourth most common cancer overall and the second most frequently diagnosed cancer among males [1]. In the United States and Europe, it is the most frequently diagnosed cancer in men [2,3]. For localized PCa, radical prostatectomy (RP) or prostate radiotherapy is the standard treatment [4,5]. In contrast, For newly diagnosed metastatic PCa patients, combination therapies with androgen deprivation therapy (ADT) are recommended, but the benefits of local therapy are still debated [6–8]. The HORRAD trial found that ADT combined with external beam radiation therapy (EBRT) delayed PSA progression more than ADT alone in patients with bone metastases and PSA > 20 ng/mL, but didn’t improve overall survival (OS) [9]. The larger STAMPEDE trial showed that prostate radiotherapy with ADT improved failure-free survival (FFS) compared to ADT alone, but didn’t affect OS in general populations [10,11]. However, subgroup analyses using the CHAARTED trial’s metastatic burden criteria found that ADT plus prostate radiotherapy significantly improved OS in patients with low metastatic burden compared to ADT alone [10–12]. These findings suggest potential survival benefits of local therapy in specific subpopulations.

Nevertheless, the role of RP, another critical local treatment modality, remains largely unexplored in metastatic PCa [13]. To address this knowledge gap, this study leverages the Surveillance, Epidemiology, and End Results (SEER) database to investigate survival outcomes associated with RP in metastatic PCa patients. Building upon prior clinical insights, we further conduct stratified analyses based on cancer characteristics.

## Patients and methods

### Data source and patient selection

The SEER database, managed by the National Cancer Institute, is a comprehensive population-based registry that collects and publishes cancer incidence and survival data, serving as a vital resource for cancer research. In this study, we used the SEER-17 dataset, which covers approximately 26% of the U.S. population. We accessed this data on March 1, 2025, and all information was anonymized and could not identify individual participants. Therefore, this study was exempted from ethical approval and informed consent

**Fig 1:**
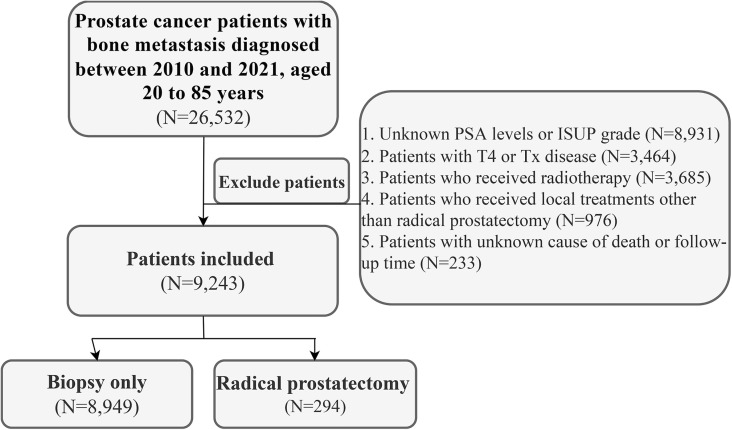
Patient selection flowchart.

We included PCa patients aged 20–85 years diagnosed between 2010 and 2021 with bone metastases at initial diagnosis. Exclusion criteria comprised: (1) unknown PSA levels or International Society of Urological Pathology (ISUP) grade; (2) clinical staging of T4 or Tx; (3) receipt of local treatments other than RP; (4) unknown cause of death or follow-up duration. Additionally, due to the lack of information regarding radiotherapy target sites in the SEER database, it was not possible to determine whether radiotherapy was directed at metastatic lesions or prostate. Therefore, patients who had received radiotherapy were also excluded from this study. The patient selection flowchart is illustrated in Fig 1.

### Statistical analysis

Patients were stratified into two groups based on local treatment: biopsy-only and RP. Baseline characteristics were compared using Pearson’s chi-square test, Wilcoxon rank-sum test, or Fisher’s exact test, as appropriate. Kaplan-Meier analysis with log-rank test was employed to estimate cancer-specific survival (CSS) and OS. The survival benefit associated with RP was assessed across different levels of PSA using restricted cubic spline (RCS) curves.

Given the baseline differences between the two groups, we used two independent methods to reduce potential bias. Firstly, we conducted 1:1 propensity score matching (PSM) to balance the baseline characteristics of the two groups and re-estimated CSS and OS using Kaplan-Meier analysis. Secondly, we performed covariate adjustment, including variables such as year of diagnosis, age, race, marital status, median household income (adjusted to 2022), residence, T stage, N stage, PSA level, ISUP grade, and chemotherapy. After confirming the robustness of the results using both methods, we noted the significant disparity in sample sizes between the two groups (8,949 in the biopsy-only group vs. 294 in the RP group). Performing PSM under such circumstances would likely result in a substantial loss of sample size, reduce statistical power, and may not fully leverage the available data. Consequently, in the subsequent analysis, we opted for covariate adjustment to better control for confounding variables and to utilize the data more efficiently.

Given prior evidence linking local therapy benefits to metastatic burden, subgroup analyses stratified by T stage, M stage, PSA level, and ISUP grade were conducted with covariate adjustment. After identifying that the survival benefit of RP over biopsy alone varied according to disease characteristics, a risk score was assigned to each patient based on disease characteristics (T stage, N stage, PSA level, and ISUP grade). Specifically, patients were assigned one point for each of the following risk factors: T3 stage, N1 stage, PSA level >72.5 ng/mL, and ISUP grade IV-V, while T0 stage, N0/Nx stage, PSA level <72.5 ng/mL, and ISUP grade I-III were assigned zero points. The total risk score was then calculated for each patient and the relationship between the total risk score and the patient’s CSS and OS was evaluated. Adjusted subgroup analyses by risk score were subsequently performed to elucidate associations between RP survival benefits and overall disease characteristics.

In this study, all statistical analyses were conducted using R software (version 4.4.2). The proportional hazards assumption in all Cox regressions in this study was verified using Schoenfeld residuals. All p-values were two-sided, and statistical significance was set at p < 0.05.

## Results

### PSA cutoff value and patient characteristics

As shown in S1 Fig, the CSS (S1A Fig) and OS (S1B Fig) benefits of RP relative to biopsy alone declined with increasing PSA levels, with confidence intervals reaching upper bounds at approximately 80 ng/ml. Considering the PSA distribution in our cohort (median value of 72.5 ng/ml), we selected 72.5 ng/ml as the cutoff value to better stratify patients and assess the differential prognostic impact of RP across PSA levels.

The baseline characteristics of the study cohort are summarized in Table 1. A total of 9,243 patients were included, with 8,949 (96.8%) receiving biopsy-only and 294 (3.2%) receiving RP. The median age of the cohort was 69 years (interquartile range [IQR], 63–76 years), with a median follow-up duration of 28 months (IQR, 13–49 months). There was a significant baseline imbalance between the two groups of patients. Compared to biopsy-only patients, those undergoing RP were younger (median age: 64 vs. 69 years), more likely to be White (79.9% vs. 75.5%), married (77.2% vs. 59.8%), have a median household income >80,000 USD (53.7% vs. 45.4%), and reside in metropolitan areas (>1 million individuals: 61.9% vs. 56.1%). Regarding disease characteristics, RP patients exhibited higher proportions of T3 stage (71.1% vs. 16.3%) and N1 stage (42.5% vs. 29.9%), but lower rates of PSA > 72.5 ng/mL (8.2% vs. 51.3%) and ISUP grade IV–V (64.3% vs. 84.3%) compared to biopsy-only patients. Additionally, RP recipients were less likely to receive chemotherapy (12.2% vs. 17.6%).

**Table 1:** Baseline characteristics of prostate cancer patients with bone metastases, 17 SEER registries, 2010-2021.

Characteristic	Local surgery			p-value	SMD
Overall, N = 9,243^1^	Biopsy only, N = 8,949^1^	RP, N = 294^1^		
Year of diagnosis				0.001^2^	
2010-2015	3,715 (40.2%)	3,624 (40.5%)	91 (31.0%)		−0.206
2016-2021	5,528 (59.8%)	5,325 (59.5%)	203 (69.0%)		0.206
Age, year	69 (63, 76)	69 (63, 76)	64 (59, 68)	<0.001^3^	−0.799
Race				0.086^4^	
White	6,995 (75.7%)	6,760 (75.5%)	235 (79.9%)		0.110
Non-White	2,191 (23.7%)	2,135 (23.9%)	56 (19.0%)		−0.122
Unknown	57 (0.6%)	54 (0.6%)	3 (1.0%)		0.041
Marital status				<0.001^2^	
Married	5,579 (60.4%)	5,352 (59.8%)	227 (77.2%)		0.415
Unmarried	2,991 (32.4%)	2,938 (32.8%)	53 (18.0%)		−0.385
Unknown	673 (7.3%)	659 (7.4%)	14 (4.8%)		−0.122
Median household income^*^				0.005^2^	
<80,000 USD	5,024 (54.4%)	4,888 (54.6%)	136 (46.3%)		−0.168
>80,000 USD	4,219 (45.6%)	4,061 (45.4%)	158 (53.7%)		0.168
Residence				0.049^2^	
<1 million individuals	4,039 (43.7%)	3,927 (43.9%)	112 (38.1%)		−0.119
>1 million individuals	5,204 (56.3%)	5,022 (56.1%)	182 (61.9%)		0.119
T stage				<0.001^2^	
T1-T2	7,572 (81.9%)	7,487 (83.7%)	85 (28.9%)		−1.208
T3	1,671 (18.1%)	1,462 (16.3%)	209 (71.1%)		1.208
N stage				<0.001^2^	
N0/Nx	6,439 (69.7%)	6,270 (70.1%)	169 (57.5%)		−0.254
N1	2,804 (30.3%)	2,679 (29.9%)	125 (42.5%)		0.254
PSA level				<0.001^2^	
<72.5 ng/ml	4,626 (50.0%)	4,356 (48.7%)	270 (91.8%)		1.576
>72.5 ng/ml	4,617 (50.0%)	4,593 (51.3%)	24 (8.2%)		−1.576
ISUP grade				<0.001^2^	
ISUP I-III	1,510 (16.3%)	1,405 (15.7%)	105 (35.7%)		0.418
ISUP IV-V	7,733 (83.7%)	7,544 (84.3%)	189 (64.3%)		−0.418
Chemotherapy				0.018^2^	
Yes	1,607 (17.4%)	1,571 (17.6%)	36 (12.2%)		−0.162
No/unknown	7,636 (82.6%)	7,378 (82.4%)	258 (87.8%)		0.162

^1^n (%); Median (Interquartile range, IQR)

^2^Pearson’s Chi-squared test

^3^Wilcoxon rank sum test

^4^Fisher’s exact test

* Adjusted to 2022

Abbreviation: RP, Radical prostatectomy; USD, United States dollar; ISUP, International Society of Urological Pathology; PSA, prostate-specific antigen

### Cancer-specific survival and overall survival

**Fig 2:**
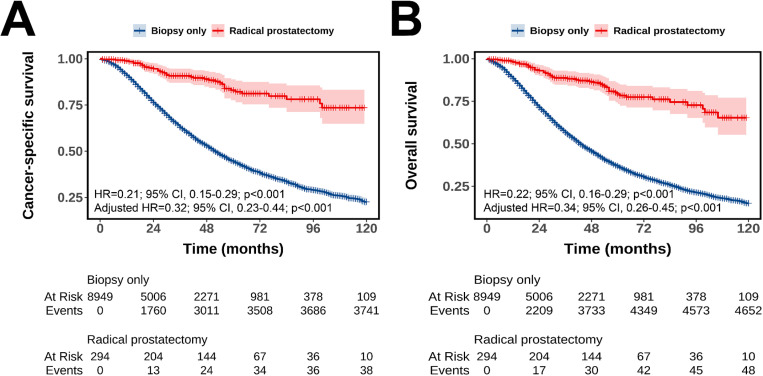
Kaplan–Meier curves comparing cancer-specific survival (A) and overall survival (B) between biopsy-alone and radical prostatectomy groups. Adjusted for covariates: year of diagnosis, age, race, marital status, median household income, residence, T stage, N stage, PSA level, ISUP grade, and chemotherapy.

As shown in Fig 2, RP was associated with superior CSS (unadjusted HR = 0.21, 95% CI: 0.15–0.29, p < 0.001; adjusted HR = 0.32, 95% CI: 0.23–0.44, p < 0.001; Fig 2A) and OS (unadjusted HR = 0.22, 95% CI: 0.16–0.29, p < 0.001; adjusted HR = 0.34, 95% CI: 0.26–0.45, p < 0.001; Fig 2B) compared to biopsy-only. Table 2 presents the CSS and OS rates at 12, 36, and 60 months. RP patients showed higher CSS rates than biopsy-only patients by 7.9% (98.9% vs. 91.0%) at 12 months, 27.9% (90.8% vs. 62.9%) at 36 months, and 38.5% (83.0% vs. 44.5%) at 60 months. OS rates for RP patients were higher by 9.8% (98.1% vs. 88.3%) at 12 months, 28.0% (88.8% vs. 56.6%) at 36 months, and 42.3% (79.2% vs. 36.9%) at 60 months.

**Table 2:** 12, 36-, and 60-month cancer-specific and overall survival rates in prostate cancer patients with bone metastasis, 17 SEER registries, 2010–2021.

Cancer type	Survival rate (95% CI, %)			p-value^*^
	12-months	36-months	60-months	
Cancer-specific survival				<0.001
Biopsy only	91.0 (90.3-91.6)	62.9 (61.8-64.1)	44.5 (43.2-45.9)	
Radical prostatectomy	98.9 (97.7-99.9)	90.8 (87.1-94.6)	83.0 (77.6-88.8)	
Overall survival				<0.001
Biopsy only	88.3 (87.6-89.0)	56.6 (55.4-57.8)	36.9 (35.6-38.2)	
Radical prostatectomy	98.1 (96.5-99.8)	88.8 (84.8-93.0)	79.2 (73.4-85.5)	

* Log-rank test

As shown in S1 Table, the baseline characteristics between the two groups of patients were well balanced after PSM. RP was still associated with significantly improved CSS (HR = 0.30, 95% CI: 0.20–0.44, p < 0.001; S2A Fig) and OS (HR = 0.33, 95% CI: 0.23–0.46, p < 0.001; S2B Fig) compared to biopsy alone. S2 Table details the CSS and OS rates at 12, 36, and 60 months post-PSM. Post-PSM, RP patients had CSS rates higher than biopsy-only patients by 2.4% (98.9% vs. 96.5%) at 12 months, 18.4% (91.3% vs. 72.9%) at 36 months, and 26.2% (83.3% vs. 57.1%) at 60 months. OS rates for RP patients were higher by 2.7% (98.1% vs. 95.4%) at 12 months, 20.0% (89.3% vs. 69.3%) at 36 months, and 27.0% (79.4% vs. 52.4%) at 60 months.

### Subgroup analysis by disease characteristics

**Fig 3:**
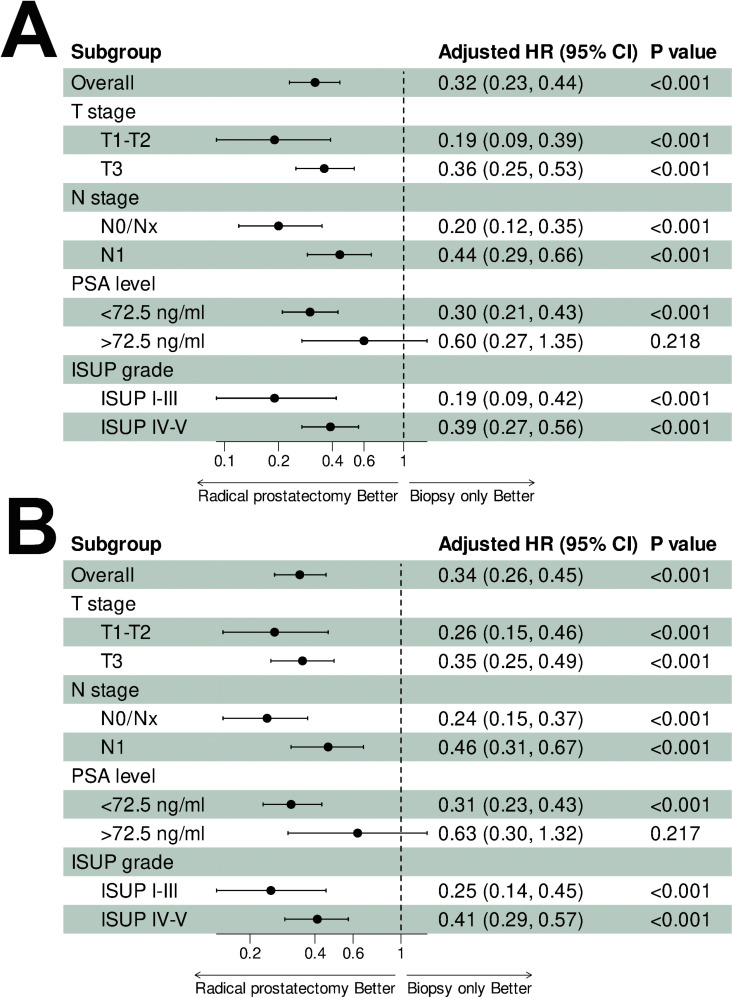
Subgroup analyses of cancer-specific survival (A) and overall survival (B) stratified by disease characteristics (T stage, N stage, PSA level, ISUP grade). Adjusted for covariates: year of diagnosis, age, race, marital status, median household income, residence, chemotherapy, and other disease characteristics (T stage, N stage, PSA level, ISUP grade) excluding the subgroup-defining variable.

Covariate-adjusted subgroup analyses stratified by T stage, M stage, PSA levels, and ISUP grade are illustrated in Fig 3. RP consistently conferred improved CSS (all adjusted HR < 1, p < 0.001, Fig 3A) and OS (all adjusted HR < 1, p < 0.001, Fig 3B) across all subgroups. Notably, though still significant, the survival benefits from RP were attenuated in patients with more advanced stage (T3 and N1) and higher grades of disease (PSA > 72.5 ng/ml and ISUP grade IV-V), suggesting that the effect of RP on survival may vary depending on disease characteristics.

### Exploratory risk score analysis

As depicted in S3 Fig, patients’ CSS (Score = 0 as reference; score = 1, HR = 2.09, p < 0.001; score = 2, HR = 2.70, p < 0.001; score = 3, HR = 2.72, p < 0.001; score = 4, HR = 2.79, p < 0.001; S3A Fig) and OS (Score = 0 as reference; score = 1, HR = 1.71, p < 0.001; score = 2, HR = 1.99, p < 0.001; score = 3, HR = 2.10, p < 0.001; score = 4, HR = 2.17, p < 0.001; S3B Fig) generally worsened with increasing risk scores (derived from T stage, N stage, PSA levels, and ISUP grade). Nevertheless, the differences in CSS and OS were not pronounced in patients with risk scores ranging from 2 to 4.

**Fig 4:**
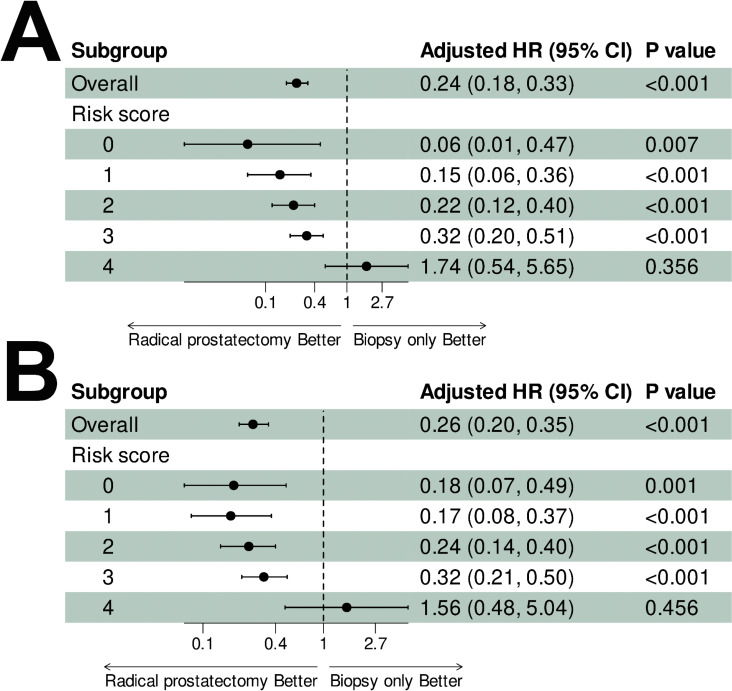
Risk score-based subgroup analyses (0–4) of cancer-specific survival (A) and overall survival (B). Adjusted for covariates: year of diagnosis, age, race, marital status, median household income, residence, and chemotherapy.

Stratified analyses based on revealed diminishing survival benefits of RP with increasing risk scores (Fig 4). Significant CSS (Fig 4A) and OS (Fig 4B) advantages were observed in patients with risk scores of 0–3. However, no significant differences were found in the highest-risk subgroup (score = 4) for CSS (adjusted HR = 1.74, 95% CI: 0.54–5.65, p = 0.356) or OS (adjusted HR = 1.56, 95% CI: 0.48–5.04, p = 0.456).

S3 Table shows the Schoenfeld residuals in all Cox regressions in this study, indicating that the proportional hazards assumption was met (all p > 0.05).

## Discussion

The survival benefits of local therapy in several metastatic malignancies have been well documented, including renal cell carcinoma, ovarian cancer, and colorectal cancer [14–17]. In PCa, 69% of panelists at the 2017 Advanced Prostate Cancer Consensus Conference recommended that radical local therapy be considered the appropriate treatment for oligometastatic PCa [18]. However, the subsequent HORRAD and STAMPEDE trials revealed that prostate radiotherapy combined with ADT failed to improve OS in unselected metastatic PCa populations, despite modest improvements in secondary endpoints such as median PSA progression time and FFS [9–11]. These results are undoubtedly disappointing, but additional analysis of STAMPEDE trail suggests that in patients with low metastatic burden, prostate radiotherapy plus ADT improves OS compared with ADT alone [10,11]. A pooled meta-analysis of HORRAD and STAMPEDE further reached similar conclusions. In unselected patients, additional prostate radiotherapy led to improved biochemical progression and FFS, but did not lead to improved OS. However, for patients with fewer than 5 bone metastases, additional prostate radiotherapy increased the 3-year survival rate by 7% [19]. Collectively, these findings underscore the metastasis burden-dependent efficacy of prostate radiotherapy, which has informed its selective integration into clinical guidelines for metastatic PCa [6–8].

In contrast, evidence supporting RP in this setting remains sparse. A meta-analysis conducted by Wang et al. showed that additional RP was associated with better OS (HR = 0.49, 95% CI: 0.44–0.55) and that RP was superior to prostate radiotherapy [20]. A recent phase II randomized trial further demonstrated survival benefits with radical local therapy (89% RP) plus ADT versus ADT alone in metastatic PCa [21]. Several retrospective studies leveraging large cancer registries, including SEER, have similarly reported favorable outcomes with RP [22–26]. However, these studies often conflated RP and radiotherapy as “local therapy,” potentially confounding efficacy assessments. Furthermore, most failed to address how disease characteristics modulate treatment effects. In addition, some SEER-based studies cannot identify the site of radiotherapy, and may mistakenly classify radiotherapy for metastatic lesions as prostate radiotherapy [24–26].

In this study, only a small minority of patients (3.2%) underwent RP. This may be largely attributable to the long-standing treatment philosophy for metastatic PCa. Traditionally, major guidelines have generally not recommended routine local prostate therapy for patients with bone metastases [6–8]. However, recent clinical trials have provided new insights, potentially prompting clinicians to increasingly recognize that local therapy might improve survival in certain bone-metastatic PCa patients and thus offering more aggressive treatment to some patients [10,11]. Notably, in our study, RP was associated with improved CSS and OS in the overall cohort, aligning with prior SEER-based reports [24–26]. Crucially, we identified heterogeneous treatment effects modulated by disease characteristics. RP conferred greater survival advantages in patients with favorable prognostic features (e.g., lower T/N stage, PSA < 72.5 ng/mL, ISUP grade I–III). Exploratory risk scoring revealed cumulative attenuation of RP benefits with worsening disease characteristics. For the highest risk patients (score = 4), RP did not improve CSS and OS compared with biopsy alone. These findings are clinically significant for identifying patients likely to benefit from RP. While database-derived survival advantages for RP have been previously reported, residual confounding in observational studies necessitates cautious interpretation. Our stratified analyses mitigate this uncertainty by delineating subgroups most likely to benefit, thereby refining patient selection criteria for future investigations. However, the small sample size in the highest risk group (score = 4) may affect the reliability of the results due to limited statistical power and potential instability of the estimates, necessitating careful interpretation of the findings in this subgroup.

It is important to note that patients with visceral metastases were excluded from this study. Visceral metastases often indicate a higher metastatic burden and more aggressive disease, which can impact the effectiveness of RP. Our research focused on patients with bone metastases, as they represent a specific subgroup where RP benefits can vary with disease characteristics. By excluding patients with visceral metastases, we better assessed RP’s impact in patients with bone metastases alone. Future research could combine cancer characteristics and genomic data to identify patients who might benefit from local therapy like RP [27,28].

## Limitations

To our knowledge, this is the first study to systematically evaluate RP survival benefits across disease characteristics in bone-metastatic PCa. Nevertheless, several limitations warrant acknowledgment: (1) Despite covariate adjustment, residual confounding inherent to retrospective SEER analyses cannot be excluded; selection bias may still be present as RP candidates were often younger and healthier. (2) The SEER database lacks detailed data on the specific regimen, duration or dose of systemic treatment (such as ADT or chemotherapy), which limits our ability to make causal inferences. (3) The lack of data on disease progression (such as PSA recurrence, imaging progression, etc.) prevents us from fully understanding the role of RP in patients with bone-metastatic PCa. (4) There is a lack of data on the location and number of bone metastases, which might have influenced the treatment response and survival outcomes. (5) The sample size of the highest-risk group (score 4) was small, so the lack of survival benefit in this group should be interpreted with caution.

Due to these limitations, future prospective cohort studies, randomized controlled trials, or other genetic study design, like Mendelian randomization studies, are warranted to validate our findings and overcome the limitations of retrospective analyses [29–32].

## Conclusion

In patients with newly diagnosed PCa with bone metastases, survival benefits associated with RP are contingent upon disease characteristics, including T stage, N stage, PSA levels, and ISUP grade. However, our findings should be interpreted with caution due to the observational nature of the study and the limitations of the database used. Future studies are warranted to refine patient selection criteria and better identify subpopulations most likely to derive clinical benefit from RP.

## Supporting information

S1 Fig Restricted cubic spline curves reveal the relationship between the advantages of radical prostatectomy over biopsy alone in cancer-specific survival (A) and overall survival (B) and prostate-specific antigen. (TIF)

S2 Fig Kaplan–Meier curves comparing cancer-specific survival (A) and overall survival (B) between biopsy-alone and radical prostatectomy groups after propensity score matching. (TIF)

S3 Fig Kaplan-Meier curves comparing cancer-specific survival (A) and overall survival (B) among patients with different risk scores (0–4). Adjusted for covariates: year of diagnosis, age, race, marital status, median household income, residence, and chemotherapy.
(TIF)

S1 Table Baseline characteristics of prostate cancer patients with bone metastases after propensity score matching, 17 SEER registries, 2010–2021. (DOCX)

S2 Table 12, 36-, and 60-month cancer-specific and overall survival rates in prostate cancer patients with bone metastasis after propensity score matching, 17 SEER registries, 2010–2021. (DOCX)

S3 Table Schoenfeld residuals for testing proportional hazards assumption. (DOCX)
