# Supplementary figures and images for: Survival benefit of radical prostatectomy in bone metastatic prostate cancer stratified by disease characteristics: A SEER-based retrospective analysis
Source: PLoS One. 2025 Jun 27;20(6):e0326429. doi: 10.1371/journal.pone.0326429 (PMC12204512; doi:10.1371/journal.pone.0326429)

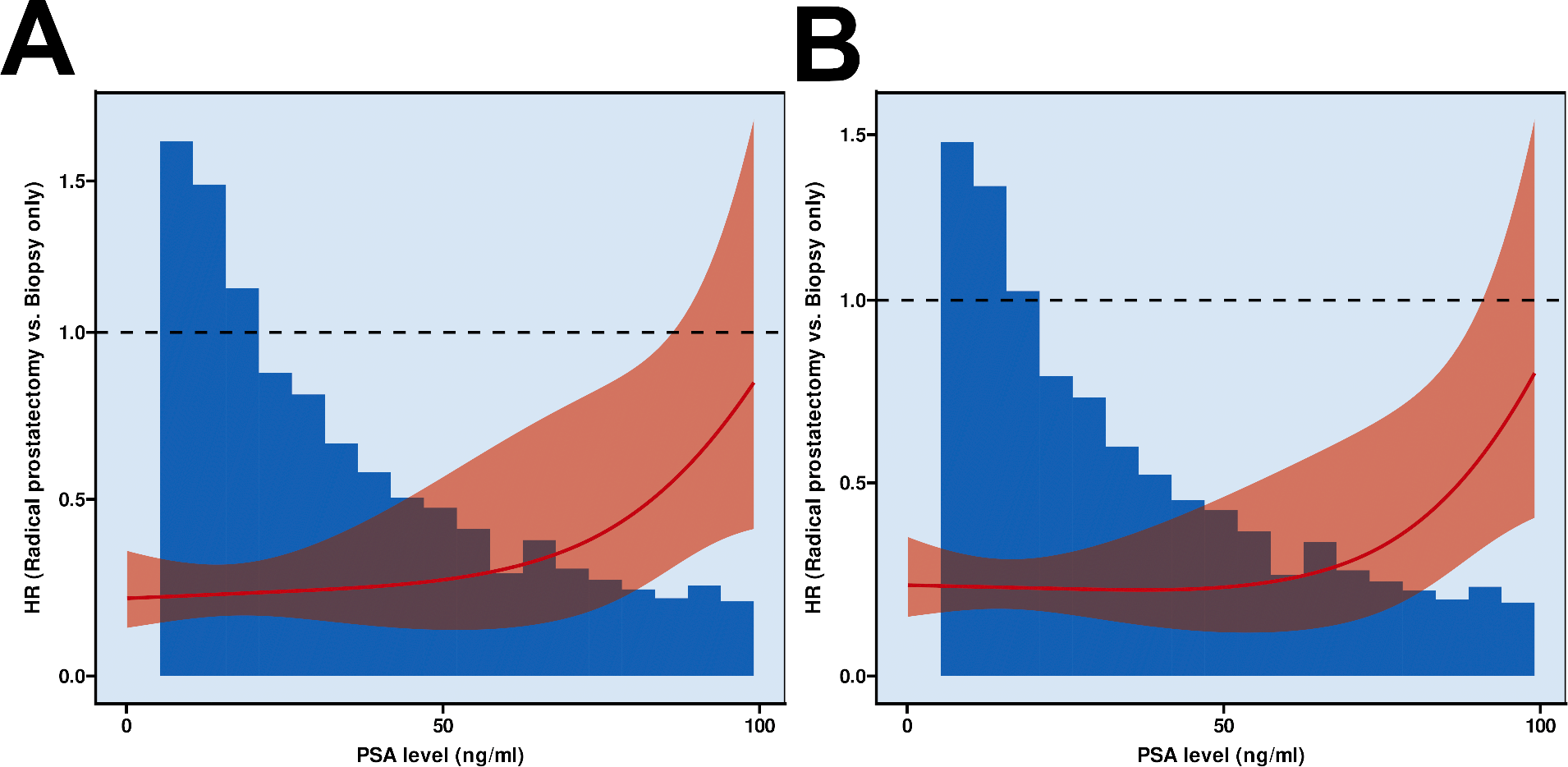

Supplement: S1 Fig — (TIF) [file pone.0326429.s001.tif]

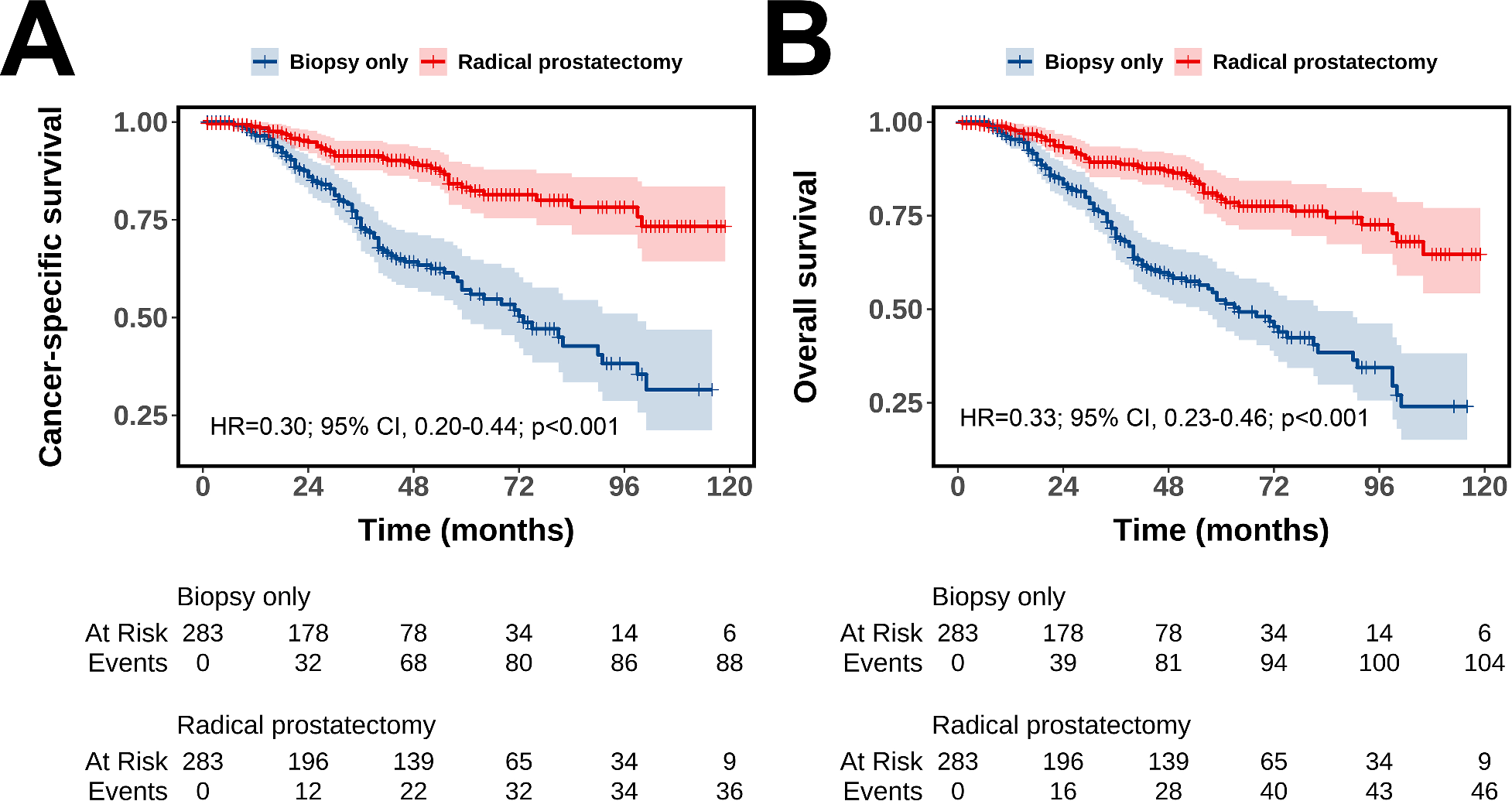

Supplement: S2 Fig — (TIF) [file pone.0326429.s002.tif]

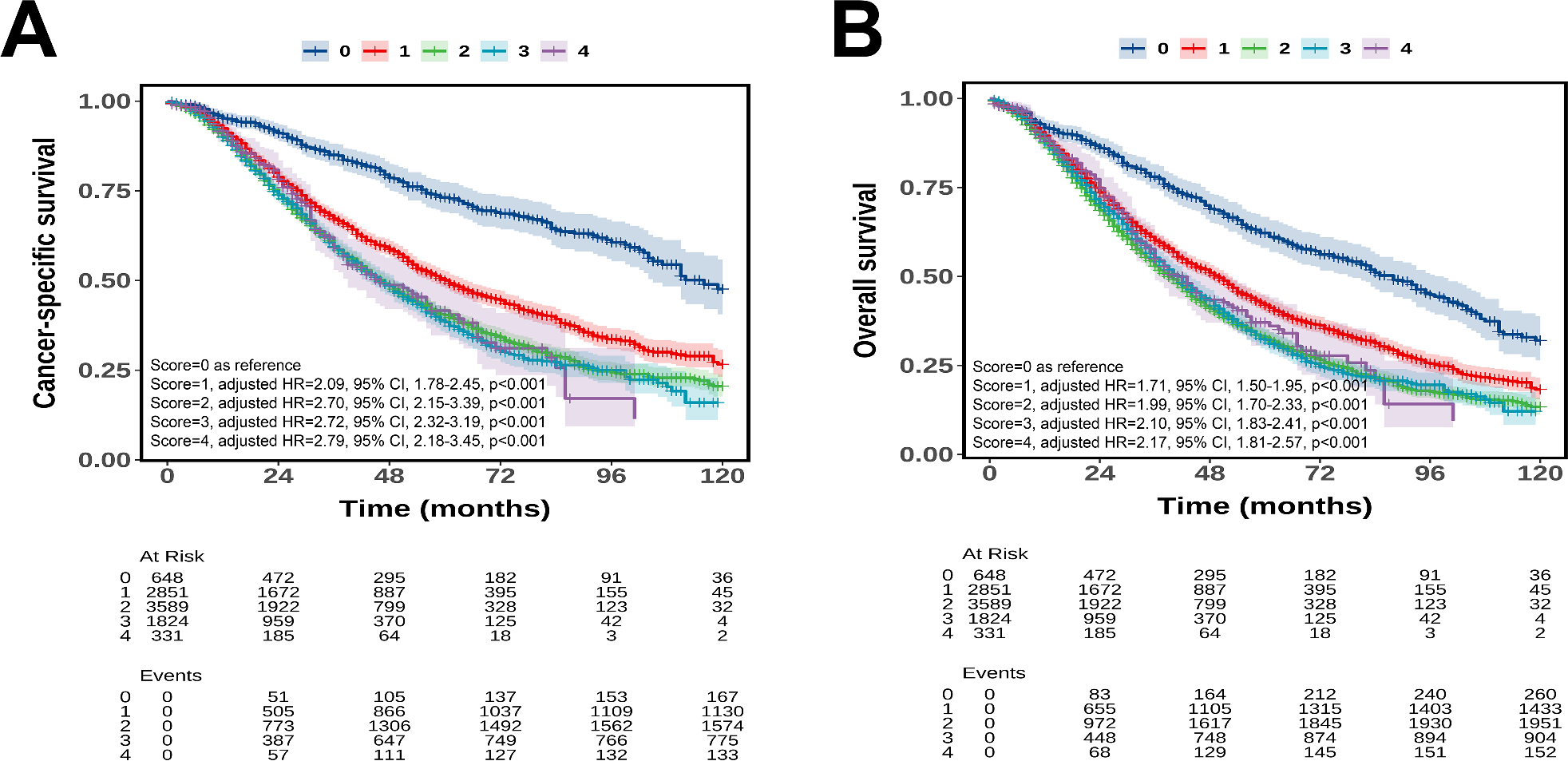

Supplement: S3 Fig — Adjusted for covariates: year of diagnosis, age, race, marital status, median household income, residence, and chemotherapy. (TIF) [file pone.0326429.s003.tif]
