# Supplementary material for: Survival benefit of radical prostatectomy in bone metastatic prostate cancer stratified by disease characteristics: A SEER-based retrospective analysis
Source: PLoS One. 2025 Jun 27;20(6):e0326429. doi: 10.1371/journal.pone.0326429 (PMC12204512; doi:10.1371/journal.pone.0326429)
Supplement: S1 Table — (DOCX) [file pone.0326429.s004.docx]

Table S1. Baseline characteristics of prostate cancer patients with bone metastases after propensity score matching, 17 SEER registries, 2010-2021

| Characteristic | Biopsy only,  N = 283^1^ | RP,  N = 283^1^ | p-value^2^ | SMD |
| --- | --- | --- | --- | --- |
| Year of diagnosis |  |  | 0.716^2^ |  |
| 2016-2021 | 198 (70.0%) | 194 (68.6%) |  | -0.031 |
| 2010-2015 | 85 (30.0%) | 89 (31.4%) |  | 0.031 |
| Age |  |  | 0.938^3^ |  |
| Median (IQR) | 64 (59, 69) | 64 (59, 69) |  | -0.014 |
| Race |  |  | 0.464^4^ |  |
| Non-White | 66 (23.3%) | 54 (19.1%) |  | -0.068 |
| White | 214 (75.6%) | 226 (79.9%) |  | 0.068 |
| Unknown | 3 (1.1%) | 3 (1.1%) |  | 0.000 |
| Marital status |  |  | 0.924^2^ |  |
| Married | 215 (76.0%) | 219 (77.4%) |  | 0.034 |
| Unmarried | 53 (18.7%) | 50 (17.7%) |  | -0.028 |
| Unknown | 15 (5.3%) | 14 (4.9%) |  | -0.017 |
| Median household income^*^ |  |  | 0.271^2^ |  |
| ˃80,000 USD | 164 (58.0%) | 151 (53.4%) |  | -0.092 |
| ˂80,000 USD | 119 (42.0%) | 132 (46.6%) |  | 0.092 |
| Residence |  |  | 0.221^2^ |  |
| ˃1 million individuals | 187 (66.1%) | 173 (61.1%) |  | -0.082 |
| ˂1 million individuals | 96 (33.9%) | 110 (38.9%) |  | 0.082 |
| T stage |  |  | 0.782^2^ |  |
| T1-T2 | 82 (29.0%) | 85 (30.0%) |  | 0.023 |
| T3 | 201 (71.0%) | 198 (70.0%) |  | -0.023 |
| N stage |  |  | 0.733^2^ |  |
| N0/Nx | 168 (59.4%) | 164 (58.0%) |  | -0.029 |
| N1 | 115 (40.6%) | 119 (42.0%) |  | 0.029 |
| PSA level |  |  | 0.879^2^ |  |
| ˂72.5 ng/ml | 260 (91.9%) | 259 (91.5%) |  | -0.013 |
| ˃72.5 ng/ml | 23 (8.1%) | 24 (8.5%) |  | 0.013 |
| ISUP grade |  |  | 0.590^2^ |  |
| ISUP I-III | 89 (31.4%) | 95 (33.6%) |  | 0.044 |
| ISUP IV-V | 194 (68.6%) | 188 (66.4%) |  | -0.044 |
| Chemotherapy |  |  | 0.622^2^ |  |
| Yes | 40 (14.1%) | 36 (12.7%) |  | -0.043 |
| No/unknown | 243 (85.9%) | 247 (87.3%) |  | 0.043 |

^1^n (%); Median (Interquartile range, IQR)

^2^Pearson's Chi-squared test

^3^Wilcoxon rank sum test

^4^Fisher's exact test

^*^Adjusted to 2022

Abbreviation: RP, Radical prostatectomy; USD, United States dollar; ISUP, International Society of Urological Pathology; PSA, prostate-specific antigen; SMD, Standardized Mean Difference
