# Supplementary material for: Survival benefit of radical prostatectomy in bone metastatic prostate cancer stratified by disease characteristics: A SEER-based retrospective analysis
Source: PLoS One. 2025 Jun 27;20(6):e0326429. doi: 10.1371/journal.pone.0326429 (PMC12204512; doi:10.1371/journal.pone.0326429)
Supplement: S2 Table — (DOCX) [file pone.0326429.s005.docx]

Table S2. 12, 36-, and 60-month cancer-specific and overall survival rates in prostate cancer patients with bone metastasis after propensity score matching, 17 SEER registries, 2010-2021

| Cancer type | Survival rate (95% CI, %) | | | p-value^*^ |
| --- | --- | --- | --- | --- |
|  | 12-months | 36-months | 60-months |  |
| Cancer-specific survival |  |  |  | <0.001 |
| Biopsy only | 96.5 (94.2-98.8) | 72.9 (66.9-79.5) | 57.1 (49.5-65.7) |  |
| Radical prostatectomy | 98.9 (97.6-100.0) | 91.3 (87.7-95.2) | 83.3 (77.8-89.2) |  |
| Overall survival |  |  |  | <0.001 |
| Biopsy only | 95.4 (92.8-98.0) | 69.3 (63.2-75.9) | 52.4 (45.1-60.9) |  |
| Radical prostatectomy | 98.1 (96.4-99.8) | 89.3 (85.3-93.4) | 79.4 (73.4-85.8) |  |

**^*^**Log-rank test
