# Supplementary material for: Survival benefit of radical prostatectomy in bone metastatic prostate cancer stratified by disease characteristics: A SEER-based retrospective analysis
Source: PLoS One. 2025 Jun 27;20(6):e0326429. doi: 10.1371/journal.pone.0326429 (PMC12204512; doi:10.1371/journal.pone.0326429)
Supplement: S3 Table — (DOCX) [file pone.0326429.s006.docx]

**Table S3. Schoenfeld residuals for testing proportional hazards assumption**

| Characteristics | Overall p-value of Schoenfeld residuals |
| --- | --- |
| Overall population |  |
| Cancer-specific survival | 0.103 |
| Overall survival | 0.098 |
| Subgroup analysis of cancer-specific survival |  |
| T1-T2 | 0.231 |
| T3 | 0.186 |
| N0/Nx | 0.321 |
| N1 | 0.099 |
| PSA level ˂72.5 ng/ml | 0.125 |
| PSA level ˃72.5 ng/ml | 0.143 |
| ISUP I-III | 0.119 |
| ISUP IV-V | 0.231 |
| Subgroup analysis of overall survival |  |
| T1-T2 | 0.241 |
| T3 | 0.136 |
| N0/Nx | 0.286 |
| N1 | 0.115 |
| PSA level ˂72.5 ng/ml | 0.136 |
| PSA level ˃72.5 ng/ml | 0.178 |
| ISUP I-III | 0.136 |
| ISUP IV-V | 0.203 |
| Subgroup analysis of cancer-specific survival based on risk scores |  |
| 0 | 0.214 |
| 1 | 0.153 |
| 2 | 0.193 |
| 3 | 0.159 |
| 4 | 0.321 |
| Subgroup analysis of overall survival based on risk scores |  |
| 0 | 0.234 |
| 1 | 0.142 |
| 2 | 0.163 |
| 3 | 0.172 |
| 4 | 0.289 |
| Cancer-specific survival analysis based on risk scores | 0.132 |
| Overall survival analysis based on risk scores | 0.173 |

Abbreviation: ISUP, International Society of Urological Pathology; PSA, prostate-specific antigen
